# Craniosynostosis, inner ear, and renal anomalies in a child with complete loss of *SPRY1* (sprouty homolog 1) function

**DOI:** 10.1136/jmg-2022-108946

**Published:** 2022-12-21

**Authors:** Rebecca S Tooze, Eduardo Calpena, Stephen R F Twigg, Felice D’Arco, The Genomics England Research Consortium, Emma L Wakeling, Andrew O M Wilkie

**Affiliations:** 1 Clinical Genetics Group, MRC Weatherall Institute of Molecular Medicine, University of Oxford, Oxford, UK; 2 Department of Radiology, Great Ormond Street Hospital for Children NHS Foundation Trust, London, UK; 3 North East Thames Regional Genetics Service, Great Ormond Street Hospital for Children NHS Foundation Trust, London, UK

**Keywords:** human genetics, genetics, musculoskeletal diseases

## Abstract

**Introduction:**

*SPRY1* encodes protein sprouty homolog 1 (Spry-1), a negative regulator of receptor tyrosine kinase signalling. Null mutant mice display kidney/urinary tract abnormalities and altered size of the skull; complete loss-of-function of Spry-1 in humans has not been reported.

**Methods:**

Analysis of whole-genome sequencing data from individuals with craniosynostosis enrolled in the 100,000 Genomes Project identified a likely pathogenic variant within *SPRY1*. Reverse-transcriptase PCR and western blot analysis were used to investigate the effect of the variant on *SPRY1* mRNA and protein, in lymphoblastoid cell lines from the patient and both parents.

**Results:**

A nonsense variant in *SPRY1,* encoding p.(Leu27*), was confirmed to be heterozygous in the unaffected parents and homozygous in the child. The child’s phenotype, which included sagittal craniosynostosis, subcutaneous cystic lesions overlying the lambdoid sutures, hearing loss associated with bilateral cochlear and vestibular dysplasia and a unilateral renal cyst, overlapped the features reported in *Spry1^−/−^* null mice. Functional studies supported escape from nonsense-mediated decay, but western blot analysis demonstrated complete absence of full-length protein in the affected child and a marked reduction in both parents.

**Conclusion:**

This is the first report of complete loss of Spry-1 function in humans, associated with abnormalities of the cranial sutures, inner ear, and kidneys.

**WHAT IS ALREADY KNOWN ON THIS TOPIC**

- Loss of sprouty homolog 1 (Spry-1) function in the mouse causes defects in the kidneys and urinary tract, altered skull size, and other phenotypes, but the consequence of loss of the human orthologue has not been described.

**WHAT THIS STUDY ADDS**

- We identifed a child homozygous for a nonsense variant affecting an N-terminal residue of *SPRY1*, causing complete loss of functional Spry-1 protein. The child's phenotype was notable for craniosynostosis and inner ear malformations, but only a minor renal abnormality. Neither parent exhibited evidence of related phenotypes.

**HOW THIS STUDY MIGHT AFFECT RESEARCH, PRACTICE OR POLICY**

- These observations suggest a mouse-human species difference in the effects of Spry-1 deficiency and question previous reports suggesting that heterozygous loss of Spry-1 is pathological. Further observations are needed to corroborate these findings.

## Introduction

Studies in *Drosophila* first identified sprouty as an important negative regulator of fibroblast growth factor receptor (FGFR) and other receptor tyrosine kinase (RTK) signalling pathways.^1^ There are four mammalian sprouty homologues (encoded by *SPRY1-SPRY4*), that in humans range in size from 288 to 319 amino acids (aa), and share three regions of homology: a canonical Cbl-tyrosine kinase-binding domain (aa 51–57), a serine-rich domain (aa 112–131) and a cysteine-rich domain (aa 181–306; numbering refers to Spry-1, see online supplemental figure 1A).^2^ Sprouty proteins lack enzymatic activity but decrease activation of the RAS-MAPK pathway, potentially by binding and sequestering the adapter protein GRB2 following sprouty phosphorylation by RTKs at key tyrosine residues, thus preventing membrane localisation of SOS, a RAS guanine exchange factor.^3^

10.1136/jmg-2022-108946.supp1 Supplementary data

Studies in mice have shown that homozygous deletion of the entire coding sequence of *Spry1* (orthologous to human *SPRY1*) affects multiple developmental processes; heterozygous mice (*Spry1* ^+/−^) are phenotypically normal. In the original report,^4^ 95% of homozygous neonates (mixed genetic background) displayed unilateral or bilateral ureter and kidney malformations, and 71% died within 5 months. These abnormalities were principally attributable to loss of negative feedback regulation of the RET RTK in the Wolffian duct,^4^ and could be phenocopied by homozygous substitution of the critical tyrosine residue (Tyr53Ala) within the Cbl-tyrosine kinase-binding domain.^4^ Notably, crossing onto a different mouse strain background (FVB) was associated with reduced penetrance of urogenital malformations in *Spry1^−/^* ^−^ animals.^5^

Subsequently, detailed studies of *Spry1* ^−/−^ mice have revealed additional defects in multiple organs and cell types, including rostral cortex,^6^ mammary gland,^7^ mesenchymal stem cells^8^ and muscle satellite cells.^9^ These phenotypes are exacerbated by adding loss-of-function (LoF) alleles of other *Spry* genes (notably *Spry2*), indicating partial functional redundancy between Spry proteins.^10^ Depending on strain background, both increased and decreased skull size were documented in *Spry1^−/−^* mice, but craniosynostosis was not reported.^11^

Bona fide disease-causing variants of human *SPRY1* have not been reported and therefore the consequence of complete LoF of the human orthologue was unknown. Here, we describe a homozygous *SPRY1* nonsense variant, identified in a patient with syndromic craniosynostosis. We show that this results in complete loss of Spry-1 and is also associated with severe defects of the inner ear and mild kidney abnormalities.

## Methods

### Genetic investigation and variant filtering of whole-genome sequencing data

Following normal routine genetic investigations, the family was enrolled into the Genomics England 100,000 Genomes Project and Illumina whole-genome sequencing (WGS) was performed on the proband and both parents.^12^ No clinically reportable variants were identified. The participant variant call format files were examined in the Genomics England Research Environment, as described.^13^ Following annotation with Ensembl Variant Effect Predictor, the variants were filtered using a gnomAD (v.2.1.1) allele frequency of ≤0.01,^14^ Combined Annotation Dependent Depletion (CADD) score ≥20 and no intersection with low complexity regions. Given the reported consanguinity, variants within homozygous regions identified using Illumina’s ROHcaller were prioritised.

### Functional analysis of cDNA and protein

Fresh blood was obtained from the proband and both parents to prepare genomic DNA and lymphoblastoid cell lines. Dideoxy-sequencing of genomic DNA was used to confirm the *SPRY1* variant. RNA was obtained from lymphoblastoid cells, following which *SPRY1* cDNA analysis (including deep sequencing) was performed by reverse transcriptase PCR (RT-PCR); protein was extracted for quantification of Spry-1 by western blotting. The antibody used to detect Spry-1 was rabbit mAb D9V6P (#13013, Cell Signaling Technology), detected using donkey anti-rabbit-horseradish peroxidase (HRP) (ab97085, abcam); anti-GAPDH-HRP (14C10, Cell Signaling Technology) was used as a loading control. Complete methods, including all primer sequences (online supplemental table 1) and PCR amplification conditions for genomic and cDNA analysis are provided in the online supplemental material.

### Targeted resequencing of *SPRY1*

A search for additional variants in *SPRY1* was undertaken by targeted resequencing of 617 individuals with previously undiagnosed craniosynostosis (online supplemental methods and online supplemental table 2).

## Results

### Case report

The index patient (II-1) is the first-born male child of consanguineous parents (half-first cousins; online supplemental figure 2A) of Indian descent. Apart from familial hypercholesterolaemia in the mother (I-2), both parents are healthy and phenotypically normal.

**Figure 1:**
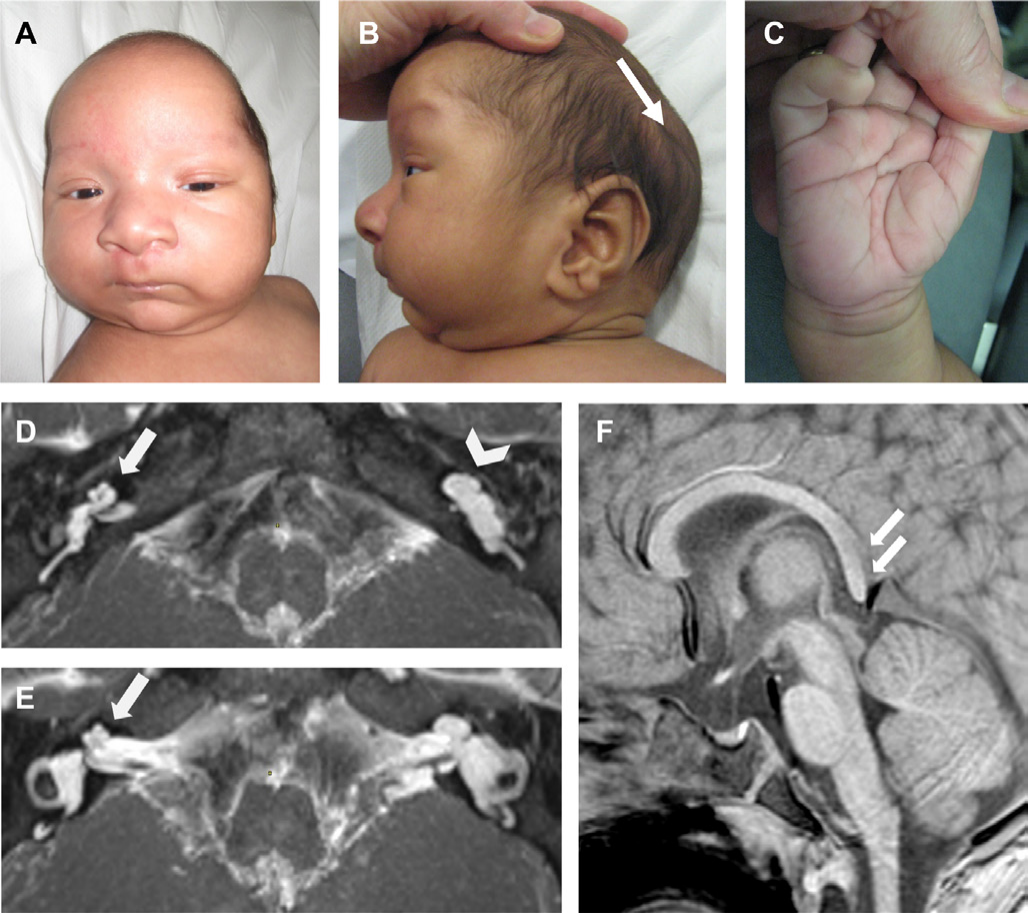
Clinical features of proband II-1 homozygous for *SPRY1* nonsense variant. (A–C) Photographs of the index patient aged 11 weeks showing scaphocephaly, turricephaly, bitemporal narrowing, hypertelorism, earlobe creases, cystic lesions overlying the lambdoid suture (B, arrow) and deep palmar crease (C). (D–F) Axial maximum intensity projection three-dimensional high-resolution T2-weighted images aged 8 months. (D–E) On his right side, he displays cochlear hypoplasia type 4 (arrows) with reasonably normal semi-circular canals. On the left, he has an incomplete partition type 1 cochlea (arrowhead), dilated vestibule with dysplastic lateral semi-circular canals. (F) Sagittal T1-weighted images showing small corpus callosum with hypoplastic splenium (double arrows).

Antenatal scans had shown increased nuchal translucency at 12 weeks and dolichocephaly from 20 weeks. Array comparative genomic hybridisation (aCGH) on an amniocentesis sample was normal. He was born at 39 weeks’ gestation and noted at birth to have scaphocephaly, turricephaly, posterior ridging of the sagittal suture and bitemporal narrowing, with tense, symmetrically positioned cystic lesions (3×1 cm) over the parietal bones bilaterally. At 12 weeks of age, his length and weight were around the 50th centile; his head circumference could not be accurately measured owing to the cystic lesions. Distinctive facial features (figure 1A, 1B) included hypertelorism, a broad base to his nose, large ears with earlobe creases, and naevus flammeus over his philtrum and central forehead. There was a capillary malformation on his upper back. In his extremities, there was soft skin on his hands (figure 1C) with deep palmar and plantar creases, and a proximally placed overriding second toe on the right. CT scan of his head (aged 10 weeks) showed sagittal synostosis and bilateral subcutaneous cystic lesions over the lambdoid sutures. Aged 4 months, he had spring-assisted cranioplasty (removed at 8 months) and scalp lesions removed (shown histologically to be dermoid cysts).

He failed his newborn hearing screen and was subsequently found to have bilateral profound sensorineural hearing loss. MRI scan of his inner ear (aged 8 months) demonstrated bilateral inner ear dysplasia. On the right, there was cochlear hypoplasia with anterior off-set markedly hypoplastic apical turn (cochlear hypoplasia 4), similar to the unwound cochlea found in branchiootorenal syndrome. The vestibule and semi-circular canals were relatively normal. On the left there was an incomplete partition type 1 malformation of the cochlea with abnormal vestibule and semi-circular canals (figure 1D, E). The VIII nerves were normal bilaterally. He underwent bilateral cochlear implantation in a two-stage procedure (aged 15 and 18 months). MRI of the brain showed a small corpus callosum, which was dysmorphic posteriorly (figure 1F).

A renal ultrasound and renogram showed a simple left renal parapelvic cyst, while the right kidney appeared normal. He has normal renal function and is normotensive with no microalbuminuria; testes and external genitalia are normal. Echocardiogram at 3 months of age showed a small atrial septal defect (which had resolved spontaneously by 4 years) and mild supravalvular pulmonary stenosis of no clinical significance.

His speech and language development were delayed, consistent with his hearing loss. However, his motor and cognitive development is normal. At 4 years of age, he uses three-word sentences and follows complex instructions. Ophthalmology review was normal, with no concerns regarding his vision.

### Molecular analysis

**Figure 2:**
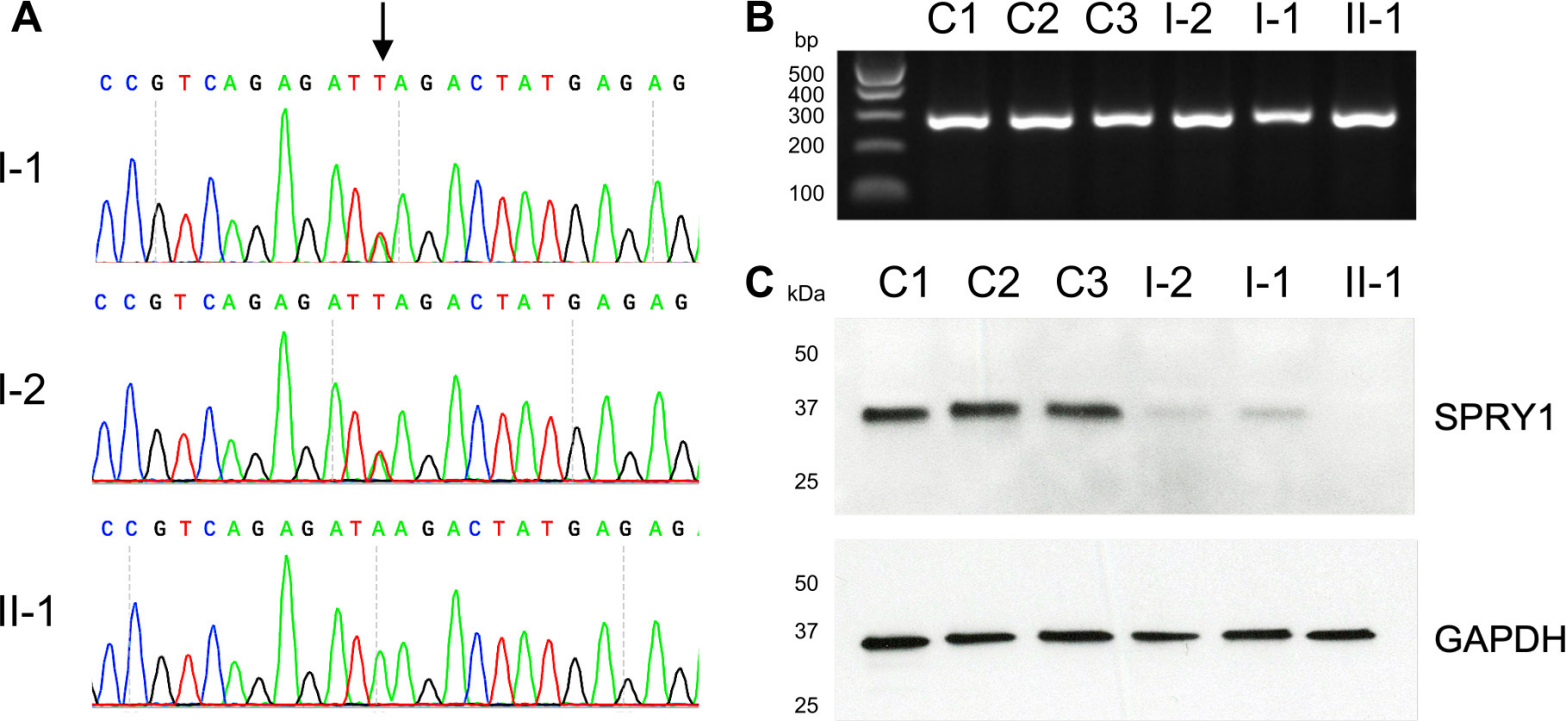
Identification of *SPRY1* nonsense variant and effect on expression of RNA and protein. (A) Dideoxy sequencing traces for each member of the pedigree. The arrow indicates the position of the c.80T>A nonsense variant in *SPRY1*. (B) RT-PCR of *SPRY1* including three unaffected controls (C1-3), the mother (I-2), father (I-1) and proband (II-1). (C) Western blot analysis using an antibody against Spry-1, targeting an epitope downstream of the stop-gain; lanes are labelled as in part B. The blots were stripped and reprobed using an antibody against GAPDH as a positive control.

Initial genetic investigations of the proband included normal postnatal aCGH and craniosynostosis gene panel testing. Analysis of WGS data from the 100,000 Genomes Project showed that his genome harboured five regions of homozygosity ≥2 Mb (online supplemental figure 2B); after filtering three homozygous variants remained, including one stop-gain (in *SPRY1*) and two predicted missense variants (in *CCDC80* and *RASSF6*). Further evaluation of the *CCDC80* and *RASSF6* homozygous variants, as well as two heterozygous de novo missense variants (in *ATXN10* and *POMP*), did not support pathogenicity of these variants (online supplemental table 3), leaving the *SPRY1* variant as the sole candidate to be prioritised for further analysis. Dideoxy-sequencing confirmed the homozygous nonsense variant (c.80T>A, encoding p.(Leu27*) in *SPRY1*), which was heterozygous in both parents (figure 2A).

The premature termination codon, p.Leu27*, arises in the last and only coding exon of *SPRY1* (online supplemental figure 1A) and is therefore predicted to escape nonsense-mediated decay (NMD).^15^ To confirm this, we undertook RT-PCR analysis of RNA extracted from lymphoblastoid cells and, in support, we identified RNA products in all three family members (figure 2B). The presence of the variant in the cDNA in approximately equal quantity to the normal allele was confirmed by deep sequencing of RT-PCR products from the parental samples, consistent with normal production of mutant RNA transcript and escape from NMD in lymphoblastoid cells (online supplemental table 4).

To assess the effect of the variant on Spry-1 protein synthesis, we undertook western blot analysis and identified a band of ~35 kDa in all healthy controls and both parents, corresponding to the expected size of Spry-1 (figure 2C). Of note, the antibody used was raised to an epitope comprising residues surrounding aa 70 of human Spry-1, located C-terminal of p.Leu27 (online supplemental figure 1A). Accordingly, the predicted truncated protein appeared entirely absent in the proband; band intensity was considerably weaker in both heterozygous parents compared with controls, consistent with loss of one copy of full length Spry-1. Results of replicate analyses and additional controls are shown in online supplemental figure 3.

## Discussion

To our knowledge, this is the first description of a patient harbouring a homozygous nonsense variant in *SPRY1*. This variant is predicted to affect all known *SPRY1* splice forms and to lead to a severely truncated Spry-1 protein that has lost all conserved features. The complete loss of full-length functional Spry-1 protein in the proband was corroborated by western blot analysis (figure 2C). The parents, who were phenotypically normal, were heterozygous for the variant and exhibited diminished amounts of full-length Spry-1 protein.

Major points of interest arising from this work are the delineation of human *SPRY1* LoF phenotypes in homozygous and heterozygous states, and comparison with the mouse null mutant. The patient shares many phenotypes previously reported in mouse *Spry1^–/–^* mutants (see ‘Introduction’ section), but with apparent differences in relative severity. Renal and ureteric anomalies, which are a major feature of the murine *Spry1^–/–^* phenotype and often lead to reduced viability, were manifested in the patient only as a unilateral simple parapelvic cyst, which did not compromise renal function. By comparison, the patient exhibited major developmental abnormalities of the inner ear, affecting both the cochlea and vestibular systems; whereas inner ears of *Spry1^–/–^* mice are normal,^16^ but manifest similar anatomical features when crossed with *Spry2* LoF alleles.^17^ Given current evidence that the renal and inner ear abnormalities are predominantly driven by dysregulated signalling through different growth factor/RTK axes (GDNF/RET and FGF/FGFR, respectively), it is possible that these signalling pathways differ in their relative sensitivity to Spry-1 inhibition in the mouse and human. A failure to repress FGF/FGFR signalling is consistent with the presence of sagittal synostosis in the patient, since dysregulated FGF/FGFR signalling is a well-documented cause of this phenotype.^18^ The patient manifested several additional phenotypes including dysmorphic facial features, bilateral dermoid cysts over the scalp and minor brain anomalies; the identification of additional cases is required to determine whether these fit into a consistent pattern.

We are aware of two previous reports of putatively pathogenic heterozygous *SPRY1* variants—a frameshift (p.(Gln6fs))^19^ in a patient with sagittal synostosis, and a stop-gain (p.(Glu79*))^20^ in a patient with Noonan syndrome (OMIM #609942). Two arguments suggest that these variants alone are unlikely to account for the associated phenotypes reported. First, the gnomAD dataset (v.2.1.1) identifies 13 individuals out of ~140 000 sequenced, including normal controls, heterozygous for LoF variants in *SPRY1*. This relatively high frequency conflicts with such alleles having highly penetrant pathogenic effects and accords with the low probability of LoF intolerance score (pLI=0). Second, in the current study, both parents were heterozygous for the LoF variant in *SPRY1* but displayed no relevant clinical phenotype. Importantly, no homozygous LoF variants are recorded in the gnomAD v.2.1.1 or v.3.1.1 datasets.

Based on similarities of the patient’s phenotype with the mouse *Spry1^–/–^* mutant and absence of other credible pathogenic variants in the patient, we propose that complete loss of Spry-1 likely accounts for the abnormal phenotypes described. Further corroboration of genotype-phenotype correlations will require the identification of additional individuals harbouring homozygous variants in *SPRY1*; however, our attempts to identify such individuals through GeneMatcher, or by sequencing of 617 patients with unsolved craniosynostosis, were unsuccessful.

In conclusion, we present the first evidence of a *SPRY1* knockout in humans. Although these observations are based on a single case study, there are substantial overlaps with the reported phenotypes in *Spry1^–/–^* mutants in mice. Together with functional evidence showing loss of full-length protein within the proband, and lack of any other identified genetic pathology, we propose that the *SPRY1* variant is likely causative.

## Data Availability

All data relevant to the study are included in the article.
