# Supplementary material for: Craniosynostosis, inner ear, and renal anomalies in a child with complete loss of SPRY1 (sprouty homolog 1) function
Source: J Med Genet. 2022 Dec 21;60(7):712–6. doi: 10.1136/jmg-2022-108946 (PMC10359576; doi:10.1136/jmg-2022-108946)
Supplement: Supplementary data [file jmg-2022-108946supp001.pdf]

Craniosynostosis, inner ear, and renal anomalies in a child with complete loss of *SPRY1* (sprouty homolog 1) function

Tooze RS, Calpena E, *et al.*

Supplemental Methods

Analysis of whole genome sequencing data

Variants were annotated with Ensembl Variant Effect Predictor[1] and a Combined Annotation Dependent Depletion (CADD)[2] score to determine the consequence and predicted pathogenicity for each variant.

Lymphoblastoid Cell Culture

Blood (1.3 ml) was mixed with an equal volume of Phosphate Buffered Saline (PBS) and added to 3 ml of Sigma Histopaque®–1077 before centrifugation at 580 x *g* for 20 min at room temperature (RT). Peripheral blood mononuclear cells (PBMCs) were collected, PBS washed, and centrifuged (RT, 350 *g*, 5 min). The supernatant was discarded, and the cell pellet washed again in PBS (RT, 180 *g*, 5 min). Epstein-Barr virus (EBV, B95-9) was added to the PBMCs and incubated at 37°C for 1.5 hours. Cells were added to phytohemagglutinin M-form (Gibco, 10576-015) and maintained in culture medium (RPMI Medium 1648 (1x) supplemented with 15% Fetal Bovine Serum (FBS; Gibco, 10082-147), 1X penicillin-streptomycin (Gibco, 15140-122) and 1X L-glutamine (Gibco, 25030-081), incubated at 37°C, 5% CO_2.

RNA extraction and cDNA synthesis

Lymphoblastoid cells were cultured and harvested by centrifugation. RNA was extracted from cell pellets using the RNeasy Mini Kit (Roche) following the manufacturer's protocol. *DNase I* (5 μl of 1U/ μl, (Sigma-Aldrich, AMPD1)) was added to 5 μl of reaction buffer (R6273) and 50 μl of eluted RNA and incubated at RT for 15 min. Stop Solution (1 μl of 50 mM EDTA) was added to DNase-treated RNA and incubated at 70°C for 10 min, before reverse transcription (RevertAid First Strand cDNA Synthesis Kit (ThermoScientific, #K1622)).

Tooze RS, *et al. J Med Genet* 2023; 60:712–716. doi: 10.1136/jmg-2022-108946

Polymerase chain reaction (PCR) of cDNA

A 2 μl sample of cDNA was diluted in a master mix containing 5 μl 5X Q5 reaction buffer, 1.25 μl each of 10 μM forward and reverse primer, 0.5 μl of dNTPs (10 mM), 5 μl of 5X Q5 GC enhancer, 0.25 μl of Q5 high fidelity enzyme and up to 25 μl of water. Samples were placed in the thermocycler under the following conditions: 98°C for 30 s, followed by 35 cycles of 98°C for 10 s, 70°C for 20 s, 72°C for 30 s, and a final elongation of 72°C for 2 min. Samples underwent dideoxy sequencing by the MRC Weatherall Institute of Molecular Medicine sequencing facility.

Next Generation Sequencing (MiSeq)

A 50ng sample of each cDNA sample was mixed with 4 μl 5X Q5 buffer, 1 μl each of 10 μM forward and reverse primer, 0.4 μl of dNTPs (10 mM), 0.2 μl of Q5 high fidelity enzyme and up to 20 μl of water. The sample was placed in the thermocycler under the following conditions: 98°C for 30 sec, followed by 30 cycles of 98°C for 10 sec, 70°C for 30 sec, 72°C for 30 sec, and a final elongation of 72°C for 8 min. The PCR product was diluted 100x and mixed with a 2 μl sample of CS barcode (2 μM, Fluidigm Access Array Barcode Library for Illumina Sequencer 100-4876) diluted in 5 μl of iProof high-fidelity master mix (BIO-RAD, 1725310) and 2 μl of nuclease-free water. Barcodes were annealed to the sample under the following conditions: 98°C for 2 min, followed by 8 cycles of 98°C for 10 sec, 60°C for 30 sec, 72°C for 30 sec and a final elongation of 72°C for 2 min. The final product was gel purified, diluted, and sequenced using a Miseq Reagent Kit v2 by the WIMM sequencing facility.

Western blot (WB)

Lymphoblastoid cells were incubated with fresh media 24 hours prior to harvest by centrifugation (300 *g*, 5 min). Lysis buffer (50 μL, Cellytic M lysis buffer (Sigma-Aldrich) containing proteases (Complete, Sigma-Aldrich) and phosphatase (PhosStop, Sigma-Aldrich) inhibitors) was added to each sample and left on ice for 30 min. The lysed samples were centrifuged at 18,000 *g* for 15 min at 4°C and the supernatant collected. Total protein concentration was quantified using a BCA Quantification Kit (ThermoScientific, 23225) and absorbance read at 562 nm (Spectra Max M2E). Samples were loaded onto polyacrylamide gels (4–20% Mini-PROTEAN® TGX™ Precast Gels, #4561094, BIO-RAD), containing 25 μg

Tooze RS, *et al. J Med Genet* 2023; 60:712–716. doi: 10.1136/jmg-2022-108946

protein sample, 6x loading dye and nuclease-free water. Samples were run for 1.5 hours at 120 V in 1x Tris-Glycine-SDS buffer before being transferred to a membrane (Immobilion®-hydrophilic polyvinylidene fluroride membrane) for 1 hour at 100 V. The membrane was blocked with 5% non-fat, blotting-grade milk (Bio-Rad) and left shaking gently for 1 hour at RT. Anti-Spry-1 (rabbit mAb D9V6P #13013, Cell Signaling Technology) diluted 1:1000 was added to the membrane and left shaking at 4°C overnight, before washing with Tween Tris-buffered saline and adding secondary donkey anti-rabbit-HRP (abcam, ab97085) for 1 hour at RT, shaking gently. SuperSignal West Pico Plus Chemiluminescent Substrate Kit (Thermo Scientific) was used to develop the signal for 5 min before imaging. The primary and secondary antibodies were removed using the restore western blot stripping buffer (Thermo Scientific) before being stained with anti-GAPDH-HRP (14C10, Cell Signaling Technology) diluted 1:10,000 at RT for 1.5 hours.

Targeted resequencing

Analysis of 617 samples with undiagnosed craniosynostosis was undertaken using IDT's hybridisation and capture protocol. DNA samples were fragmented following the Swift 2S™ Turbo v2 DNA Library Kit protocol and analysed using broad-range Qubit and D1000 TapeStation reagents, ensuring an average fragment size of 330bp. The prepared libraries were pooled to a total of 6 μg of DNA and up to 40 samples per hybridisation capture reaction. The hybridisation reactions were carried out at 65°C for 16 hours. After hybridisation, the pooled libraries were washed and post-capture PCR was performed, following manufacturer's protocol (IDT xGen hybridization capture of DNA libraries for NGS target enrichment) for a panel containing 2054 probes. The amplified capture reactions were washed with beads and quantified and validated using high-sensitivity (HS) qubit reagents and HS D1000 TapeStation before next generation sequencing analysis using Miseq. Variants were analyses using amplimap software.[3]

Supplemental Table 1: List of primers

Primer Name	Sequence (5'-3')	Length	Tm	GC%	Assay
SPRY1_Leu27_F	TGCCAGGTTTCCACTGATT	19	57	47	Sanger Sequencing
SPRY1_1R	GTGTGTCTGTGCTCGTAGTT	20	57	50.0	Sanger Sequencing
SPRY1_Ex2-3_Junc_F	GAAAAGGGATTTCAGATGCATGCCAGG	27	60	48	RT-PCR
SPRY1_Ex3_R	CATGCTTTTCTTGTCTTGGTGCTGTC	26	61	46	RT-PCR
SPRY1_Ex3_CS_FW	ACACTGACGACATGGTTCTAACAGAA AAGGGATTTCAGATGCATGCCAGG	51	68.2	45	NGS
SPRY1_Ex3_CS_RV	TACGGTAGCAGAGACTTGGTCTCATG CTTTTCTTGTCTTGGTGCTGTC	48	68	48	NGS

Supplemental Table 2: Coordinates of the capture probes used in the targeted resequencing analysis

**Chromosome**	**Start**	**End**	**Probe ID**
chr4	123401501	123401621	788227_47098358_Target303_SPRY1_ENST00000610581.4_1
chr4	123401561	123401681	788227_47098358_Target303_SPRY1_ENST00000610581.4_2
chr4	123401621	123401741	788227_47098358_Target303_SPRY1_ENST00000610581.4_3
chr4	123401681	123401801	788227_47098358_Target303_SPRY1_ENST00000610581.4_4
chr4	123401741	123401861	788227_47098358_Target303_SPRY1_ENST00000610581.4_5
chr4	123401801	123401921	788227_47098358_Target303_SPRY1_ENST00000610581.4_6
chr4	123401861	123401981	788227_47098358_Target303_SPRY1_ENST00000610581.4_7
chr4	123401921	123402041	788227_47098358_Target303_SPRY1_ENST00000610581.4_8
chr4	123401981	123402101	788227_47098358_Target303_SPRY1_ENST00000610581.4_9
chr4	123402041	123402161	788227_47098358_Target303_SPRY1_ENST00000610581.4_10
chr4	123402101	123402221	788227_47098358_Target303_SPRY1_ENST00000610581.4_11
chr4	123402161	123402281	788227_47098358_Target303_SPRY1_ENST00000610581.4_12
chr4	123402221	123402341	788227_47098358_Target303_SPRY1_ENST00000610581.4_13
chr4	123402281	123402401	788227_47098358_Target303_SPRY1_ENST00000610581.4_14
chr4	123402341	123402461	788227_47098358_Target303_SPRY1_ENST00000610581.4_15
chr4	123402401	123402521	788227_47098358_Target303_SPRY1_ENST00000610581.4_16
chr4	123402461	123402581	788227_47098358_Target303_SPRY1_ENST00000610581.4_17
chr4	123402521	123402641	788227_47098358_Target303_SPRY1_ENST00000610581.4_18

Tooze RS, *et al. J Med Genet* 2023; 60:712–716. doi: 10.1136/jmg-2022-108946

Supplemental Table 3: Variants identified after filtering by allele frequency and CADD score

Gene	Chr	Pos[a]	Nucleotide and amino acid change	Mode of inheritance	Allele Frequency (AF)[b]	dbSNP	CADD score (PHRED)	Reported Phenotypes	Reason discounted
CCDC80	3	112609989	NM_199511.2: c.2414C>T; p.(Ala805Val)	Recessive	0.008783	rs56683778	31	Colorectal carcinoma	60 reported homozygous A805V variants in gnomAD V2.1.1
RASSF6	4	73587902	NM_177532.5: c.320A>G; p.(Tyr107Cys)	Recessive	-	rs776608439	25.5	Multiple cancers	Multiple amino acids tolerated at this position.
SPRY1	4	123401671	NM_001258038.2: c.80T>A; p.(Leu27*)	Recessive	-	-	35	Craniofacial malformation Kidney and urinary tract malformation	-
POMP	13	28664562	NM_015932.6: c.155A>G; p.(Glut52Gly)	*De novo*	-	-	31	Keratosis linearis with ichthyosis congenita and sclerosing keratoderma; proteasome-associated autoinflammatory syndrome 2	Associated disorders arise from indels. Described phenotypes are not consistent with our patient.[4]
ATXN10	22	45700287	NM_013236.4: c.397C>T; p.(Arg133Cys)	*De novo*	0.000024	-	28.1	Spinocerebellar ataxia	6 other reported heterozygous variants in gnomAD. Atxn10 heterozygous mice show no overt phenotype.[5]

[a]Genome GRCh38
[b]gnomAD AF taken from V2.1.1 (combined exome and genome data). AF for *de novo* variants was set at below 0.001, whereas AF for recessive variants was set at less than 0.01.

Tooze RS, *et al. J Med Genet* 2023; 60:712–716. doi: 10.1136/jmg-2022-108946

Supplemental Table 4: Deep sequencing of cDNA from both parents and the affected child

Family Member	Reference Allele	Alternative Allele
**Mother**	6126/12692 = 48%	6565/12692 = 52%
**Father**	11841/22180 = 53%	10337/22180 = 47%
**Proband**	5/21937 = 0.02%	21930/21937 = 100%

Tooze RS, *et al. J Med Genet* 2023; 60:712–716. doi: 10.1136/jmg-2022-108946

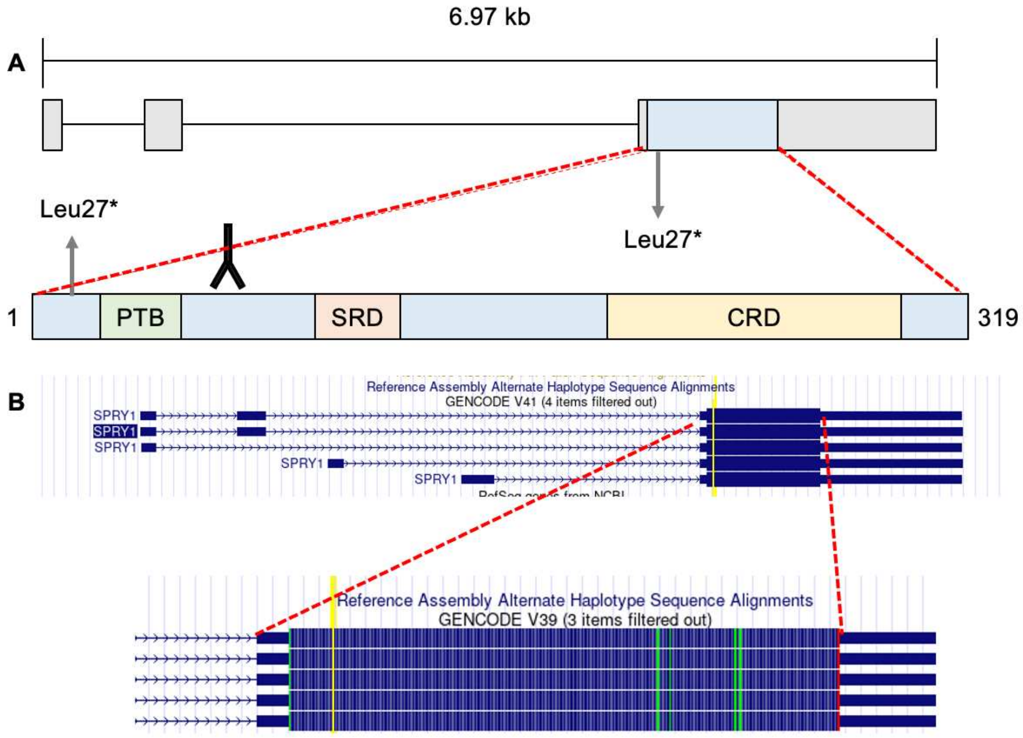

Supplemental Figure 1: *SPRY1* exon structure and predicted consequence of p.(Leu27)* nonsense variant. (A) Schematic of *SPRY1* with the coding region marked in blue and the non-coding exons in grey. Below, encoded protein showing conserved domains (PTB, phosphotyrosine-binding domain; SRD, serine-rich domain; CTD, cysteine-rich domain). The position of the antibody in the translated protein is indicated (inverted Y). (B) Screenshot from the UCSC web browser (https://genome.ucsc.edu/) showing that all transcripts of *SPRY1* would be affected by the p.(Leu27*) variant (position marked by the yellow line). Below, the coding exon has been zoomed in to identify any further methionine residues (highlighted in green), which all reside downstream of p.(Leu27).

Tooze RS, *et al. J Med Genet* 2023; 60:712–716. doi: 10.1136/jmg-2022-108946

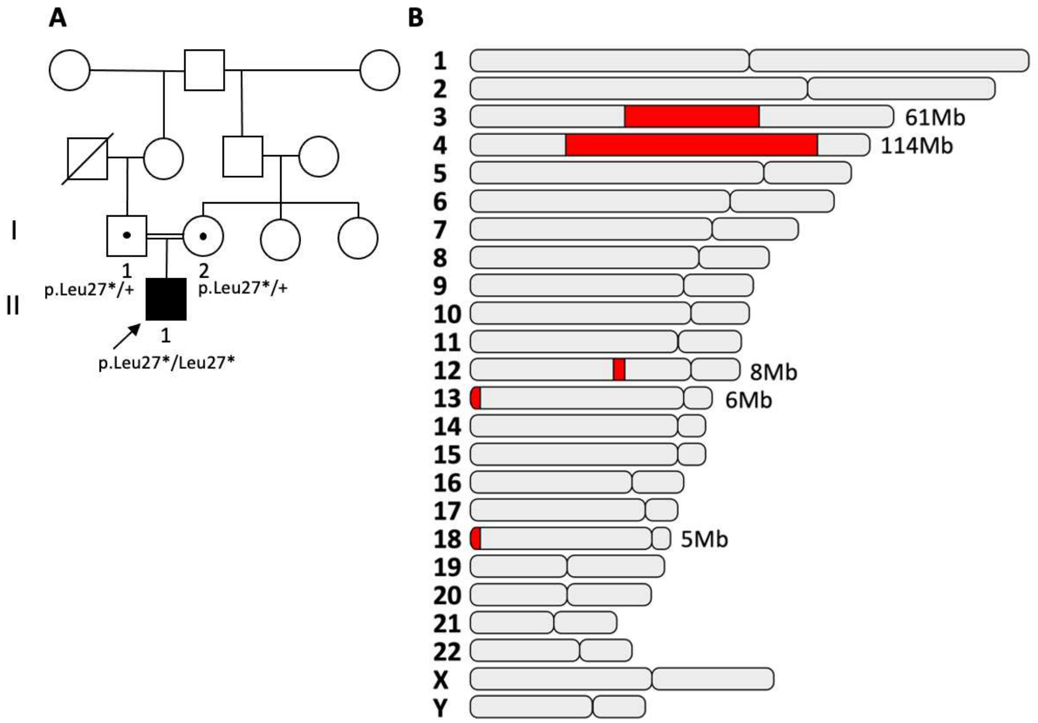

Supplemental Figure 2: Family pedigree and homozygosity analysis of WGS data from the proband II-1. (A) Pedigree figure illustrating both heterozygous parents with known consanguinity and the index patient, homozygous for p.(Leu27*) in SPRY1. (B) The output from ROHcaller is depicted in a schematic showing each chromosome, with regions of homozygosity larger than 2 Mb highlighted in red. *SPRY1* resides within the largest region of homozygosity (114 Mb) located on chromosome 4.

Tooze RS, *et al. J Med Genet* 2023; 60:712–716. doi: 10.1136/jmg-2022-108946

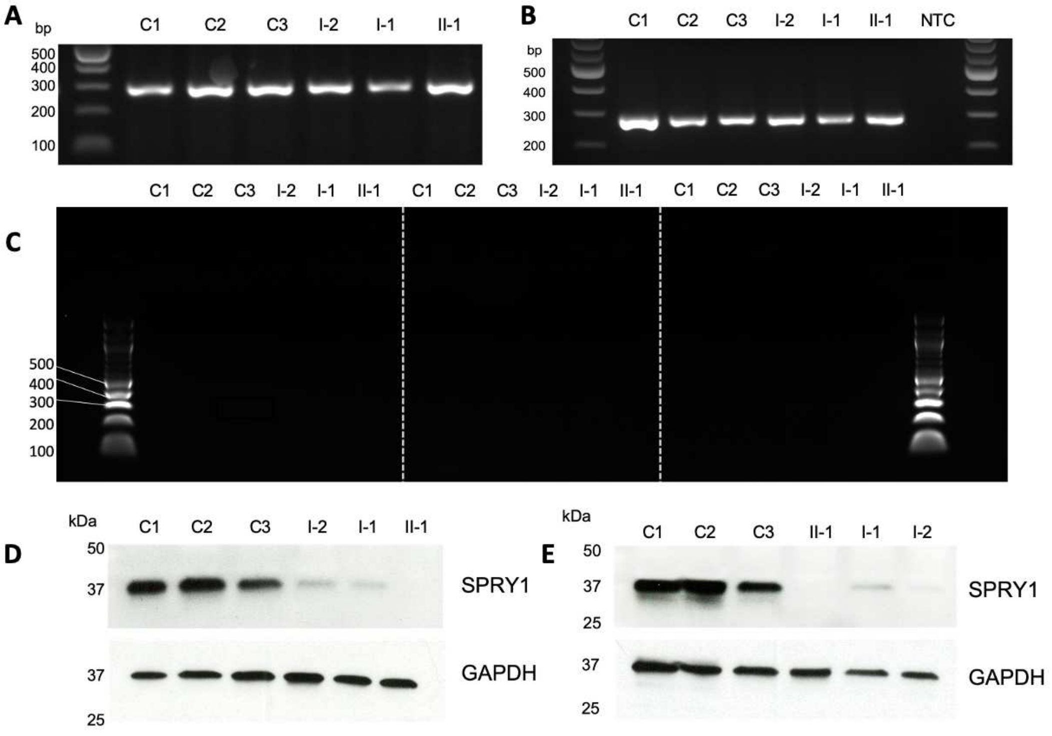

Supplemental Figure 3: Additional replicates of functional studies. (A-B) Two additional biological replicates of the RT-PCR including a negative no-template control (NTC). C1-3 = control 1-3, I-2 = mother, I-1 = father, II-1 = proband. (C) All no reverse-transcriptase controls (24 samples, including three repeats of C1-3, I-1, I-2, and II-1) were amplified using *SPRY1* primers and run on a 2% ethidium bromide gel. (D-E) Two further repeats of the western blot analysis alternating sample position.

REFERENCES

1. McLaren W, Gil L, Hunt SE, et al. The Ensembl Variant Effect Predictor. *Genome Biol* 2016;17(1):122.
2. Rentzsch P, Witten D, Cooper GM, et al. CADD: predicting the deleteriousness of variants throughout the human genome. *Nucleic Acids Res* 2019;47(D1):D886-D94.
3. Koelling N, Bernkopf M, Calpena E, et al. Amplimap: a versatile tool to process and analyze targeted NGS data. *Bioinformatics* 2019;35(24):5349-50.
4. Poli MC, Ebstein F, Nicholas SK, et al. Heterozygous Truncating Variants in POMP Escape Nonsense-Mediated Decay and Cause a Unique Immune Dysregulatory Syndrome. *Am J Hum Genet* 2018;102(6):1126-42.
5. Wakamiya M, Matsuura T, Liu Y, et al. The role of ataxin 10 in the pathogenesis of spinocerebellar ataxia type 10. *Neurology* 2006;67:607-13.
